# Epifaunal invertebrate assemblages associated with branching Pocilloporids in Moorea, French Polynesia

**DOI:** 10.7717/peerj.9364

**Published:** 2020-06-19

**Authors:** Chiara Pisapia, Jessica Stella, Nyssa J. Silbiger, Robert Carpenter

**Affiliations:** 1 Department of Ocean Science and Hong Kong Branch of the Southern Marine Science and Engineering Guangdong Laboratory (Guangzhou), The Hong Kong University of Science and Technology, Kowloon, Hong Kong; 2 Department of Biology, California State University, Northridge, CA, USA; 3 Great Barrier Reef Marine Park Authority, Townsville, QLD, Australia

**Keywords:** Coral-associated invertebrates, Partial mortality, Current water flow, Biodiversity, Decapoda, Trapezia, Coral reefs, Moorea, Branching corals, Habitat availability

## Abstract

Reef-building corals can harbour high abundances of diverse invertebrate epifauna. Coral characteristics and environmental conditions are important drivers of community structure of coral-associated invertebrates; however, our current understanding of drivers of epifaunal distributions is still unclear. This study tests the relative importance of the physical environment (current flow speed) and host quality (e.g., colony height, surface area, distance between branches, penetration depth among branches, and background partial mortality) in structuring epifaunal communities living within branching *Pocillopora* colonies on a back reef in Moorea, French Polynesia. A total of 470 individuals belonging to four phyla, 16 families and 39 genera were extracted from 36 *Pocillopora* spp. colonies. Decapods were the most abundant epifaunal organisms (accounting for 84% of individuals) found living in *Pocillopora* spp. While coral host characteristics and flow regime are very important, these parameters were not correlated with epifaunal assemblages at the time of the study. Epifaunal assemblages associated with *Pocillopora* spp. were consistent and minimally affected by differences in host characteristics and flow regime. The consistency in abundance and taxon richness among colonies (regardless of habitat characteristics) highlighted the importance of total habitat availability. With escalating effects of climate change and other localized disturbances, it is critical to preserve branching corals to support epifaunal communities.

## Introduction

Coral reefs are highly productive ecosystems and provide shelter and food for a vast number of organisms (Glynn & Enochs, 2011; Pratchett et al., 2011; Carvalho et al., 2019). Branching scleractinian corals can harbour high abundances of invertebrate epifauna, mostly from the phyla Arthropoda and Mollusca (Stella, Jones & Pratchett, 2010; Stella et al., 2011; Enochs, 2012), that usually form a symbiotic relationship with the coral host (Knudsen, 1967; Glynn, 1983; Stimson, 1990).

The complex architecture of branching corals provide shelter as well as food in the form of coral tissue, mucus, and associated detritus to the taxa that live within them (Castro, 1988). In turn, many branching corals are reliant upon invertebrates that live among their branches for protection from predators (Pratchett, 2001; McKeon et al., 2012; McKeon & Moore, 2014) and cleaning of sediment deposits (Stewart et al., 2006), thus forming mutually beneficial associations (Glynn, 1980, 1987; Leray et al., 2012). Coral crabs of the genus *Trapezia*, for instance, protect branching corals from corallivorous crown-of-thorns sea stars and can reduce the negative effects vermetid snails may have on coral growth and overall survival (Shima, Osenberg & Stier, 2010; Stier et al., 2010). Although some symbionts could be harmful (i.e., corallivorous snails), many are beneficial and these are the most abundant (McClanahan, 1994).

Approximately 870 species of invertebrates associate with corals (Stella et al., 2011), but the true diversity may be much higher (Carvalho et al., 2019) and vary in relation to reef habitat diversity and the topographic complexity provided by scleractinian corals (Sale, 1991; Wilson, Graham & Polunin, 2007; Graham & Nash, 2013). Light attenuation and availability can vary greatly within a colony, thus providing a variety of light microenvironments for exosymbionts (Kaniewska et al., 2011). Invertebrates most reliant upon branching corals are vulnerable to severe impacts if declines in coral cover and abundance occur (Stella, Munday & Jones, 2011; Enochs, 2012). Coral mortality can lead to a reduction in the abundance of epifaunal taxa, but it also depresses individual fitness of invertebrates with consequences for their recruitment and population persistence (Pratchett et al., 2004; Stella, Munday & Jones, 2011; Stella et al., 2014).

The characteristics of coral colonies and environmental conditions are important drivers of community structure of reef-associated invertebrates (Klumpp, McKinnon & Mundy, 1988; Kaandorp, 1999; Vytopil & Willis, 2001; Kane et al., 2009; López-Pérez et al., 2017; Counsell et al., 2018) and of reef fish communities (Jones et al., 2004; Feary et al., 2007). Habitat selection and availability determine spatial distribution and abundance of epifaunal assemblages on coral reefs. Invertebrates may prefer complex habitat structures that prioritize refuge from predation (Stella et al., 2011). The density and richness of organisms harbored in coral colonies with variable and complex branch growth forms, exemplified by many Pocilloporids, are often high (Vytopil & Willis, 2001; Kane et al., 2009; Stella, Jones & Pratchett, 2010; Stella et al., 2011). Similarly, high water flow may enhance richness and diversity of epifaunal invertebrates by increasing nutrient supply and particulate matter relied upon as food by coral-dwellers (Kaandorp, 1999).

Since flow regime varies greatly both at the scale of a single coral colony and entire reef, epifaunal assemblages can exhibit a range of responses to different flow conditions, depending on their tolerance to hydrodynamic processes (Kaandorp, 1999; Hench & Rosman, 2013). For instance, the reef crest is a shallow wave-exposed habitat often exposed to high flow regime, which may strongly favor organisms that are mechanically and physiologically adapted (Madin et al., 2013; Pisapia, Anderson & Pratchett, 2014). There is evidence that wave energy may play a significant role in structuring cryptic coral reef fish communities with calmer sites showing higher diversity and abundance of small cryptic fishes (Depczynski & Bellwood, 2005). The morphology of coral colonies can also vary greatly with different flow regimes (Madin & Connolly, 2006; Madin et al., 2014), thereby affecting the abundance and taxon richness of epifaunal communities associated with these colonies (Kaandorp, 1999; Kane et al., 2009). Importantly, flow is intricately involved in dictating settlement processes affecting dispersal and supply of larvae (Willis & Oliver, 1990). Larval concentration and delivery rates are strongly affected by current flow speed (Cowen & Castro, 1994). These pre-settlement factors strongly influence how epifaunal communities aggregate to corals.

Colony size is another important factor in driving distribution and persistence of epifaunal assemblages. It is expected that with increasing colony area the amount of habitat available increases and as a result the number of species present increase (Simberloff, 1972). For instance, both the number and size of crabs associated with branching Pocilloporid colonies on shallow reefs are positively related to colony size (Castro, 1978). Some invertebrates such as *Trapezia* crabs move to a larger host colony when the rate at which they ingest coral mucus becomes detrimental to the colony (Castro, 1978). *Trapezia* spp. are territorial and only one breeding pair typically occupies a single host colony regardless of colony size (Preston, 1973; Stier et al., 2012). Some invertebrates such as decapod crustaceans have an asymptotic relationship with colony size at about 6,000 cm^3^ so new species are not added with increasing colony area (Abele & Patton, 1976). Many studies have investigated the relationship between invertebrates assemblages and their coral host (Stella, Jones & Pratchett, 2010; Holbrook, Schmitt & Brooks, 2011; Leray et al., 2012; Head et al., 2015; Britayev et al., 2017; Counsell et al., 2018), and, in particular, many studies documented that colony size is a key driver in invertebrates community metrics (Abele & Patton, 1976; Julian Caley, Buckley & Jones, 2001; Leray et al., 2012). However, how epifaunal assemblages scale with the coral host remains partially unclear. For example, the abundance and size of invertebrates such as *Tetralia* crabs are independent of live coral surface area (Vytopil & Willis, 2001), indicating that the relationship between colony size and epifaunal assemblages may vary. Understanding under what contexts invertebrate abundance and taxon richness scale with colony size is critical, as loss of larger colonies may greatly enhance the risk of extirpation or extinction of coral-dwellers.

Previous studies have documented the importance of partial mortality (e.g., loss of live polyps) in driving epifaunal assemblages (Stella, Jones & Pratchett, 2010; Leray et al., 2012). Dead coral colonies and colonies with high partial mortality may harbor higher diversity of invertebrates, especially obligate coral dwelling organisms (Stella, Jones & Pratchett, 2010; Enochs, 2012; Leray et al., 2012; Head et al., 2015). With declining live tissue cover, new microhabitats within the colony are formed allowing for other species to utilize new available resources (Stella, Jones & Pratchett, 2010). Partial mortality and colonization of the dead skeleton by algae may thus favor a mixed community composed of both coral obligate and non-obligate species and an increase in species diversity. Leray et al. (2012) documented significant changes in decapod communities with partial mortality, observing an increase in species diversity with increasing tissue loss. However, the increase in partial mortality also resulted in a shift from coral obligate to non-obligate decapods species. This study aimed to refine previous findings and improve understanding of the role of partial mortality in structuring epifaunal assemblages.

In recent years, unprecedented degradation of coral reefs habitats due to global warming has occurred throughout the tropics (Heron et al., 2017; Hughes et al., 2017). Habitat degradation has not been uniform on reefs, but rather it correlated with patterns of thermal stress which varied according to reef position and depth (Hughes et al., 2017, 2018, 2019). Flow regime can dramatically influence the outcome for the same species of corals located on different parts of the reef (West & Salm, 2003). Since flow may contribute to patchiness in habitat degradation, it is expected that the outcome of coral-associated invertebrates could also vary with flow. Here, we investigated how current flow speed and host characteristics such as colony height (which may be the most likely feature to interact with differential flow) and partial mortality relate to density and taxon richness of epifaunal assemblages associated with Pocilloporids on a reef flat in Moorea, French Polynesia. This study could aid in a better understanding of consequences of habitat degradation and loss of biodiversity on coral reefs.

## Materials and Methods

**Figure 1:**
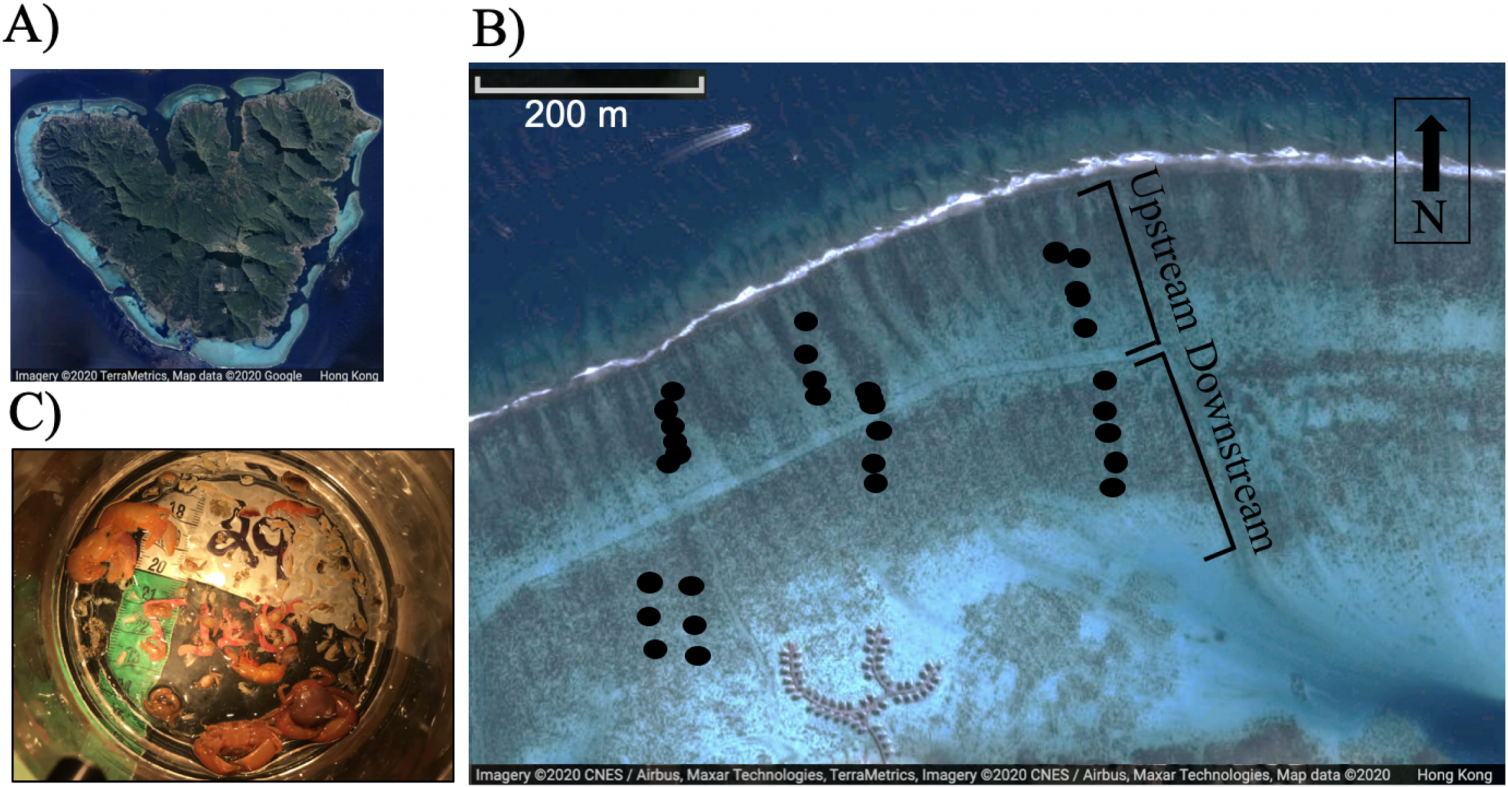
(A) Image of Moorea, (B) relative position of study corals on the reef flat, each dot represents a colony, and (C) typical epifaunal assemblage observed in association with one colony of *Pocillopora* spp. in Moorea. A total of 18 colonies were included in the upstream and downstream category respectively (*n* = 36 adult colonies). Images (A) and (B) were created using Google Maps/ Google Earth: map data © 2020 CNES/Airbus, Maxar Technologies, TerraMetrics.

We investigated epifaunal invertebrate assemblages associated with colonies of the branching *Pocillopora* spp., in Moorea, French Polynesia in January 2018. Twelve colonies of *Pocillopora* spp. were haphazardly collected between the reef crest and shore along three reef transects (~400 m) in ~2 m depth (*n* = 36 adult colonies), where flow regime has been extensively described through time series analyses of the Moorea Coral Reef Long Term Ecological Research program (MCR-LTER; http://mcr.lternet.edu; Hench, Leichter & Monismith, 2008; Hench & Rosman, 2013). Flow across the reef flat on the north shore of Moorea is unidirectional from the crest towards back reef and it has been shown to be relatively uniform across the whole flat (Hench, Leichter & Monismith, 2008; Hench & Rosman, 2013). To ensure that differences in epifaunal assemblages were representative of assemblages on the north shore of Moorea, three back reef sites were haphazardly chosen to collect coral colonies (Fig. 1). The presence of a boating channel running through the study sites may have influenced some of the variables measured, either by disrupting natural flow patterns or influencing animal behavior, particularly that of predatory fishes near the channel which may influence epifaunal communities. Consequently, the colonies collected directly adjacent to the channel were examined for any persistent differences from the rest of the dataset using an ANOVA. However, no differences were found.

*Pocillopora* spp. are some of the most common branching corals in the back reef community of Moorea (Edmunds, Leichter & Adjeroud, 2010). Since morphological features are unreliable indicators of species (Edmunds et al., 2016; Johnston et al., 2017), colonies were identified at genus level based on genera specific morphological features (Veron, 2000; Edmunds et al., 2016; Johnston et al., 2017).

We tested the hypotheses that flow speed, colony characteristics, and partial mortality affected the community structure of the epifaunal assemblage associated with *Pocillopora* spp. by haphazardly selecting different colonies across a flow speed and wave exposure gradient (sampling from reef crest to the shore). The north shore back reef, where the study was conducted, is characterized by wave-driven circulation (Hench, Leichter & Monismith, 2008). Waves drive flow over the reef crest, through the lagoon, and back out through a pass (Hench, Leichter & Monismith, 2008). Flow was measured near the crest and near the shore at the MCR-LTER site during the study for six consecutive days. Three-dimensional water velocity was measured using a Nortek (Boston, MA) Aquadopp acoustic doppler profilers (ADP) deployed upstream near the reef crest and another deployed downstream (ca 500 m from the reef crest). Flow speed was measured in 10-cm bins from 20 cm above the benthos to the water surface at 1-min intervals (accuracy ±1% of measured value ±0.5 cm/s). Because flow was measured at one upstream and downstream site, it was not possible to investigate whether there was a relationship between actual flow velocity and invertebrate community composition. Conversely, this study investigated whether the position of colonies upstream versus downstream (Fig. 1) may correlate to epifaunal abundance and diversity. The colonies ranged from 67 m to 458 m away from the crest.

To characterize the mobile epifaunal invertebrates, each colony was immediately placed in a plastic bag and transported in an individual shaded bucket to the Richard B. Gump South Pacific Research station. Colonies were processed to investigate epifaunal assemblages and host characteristics immediately after collection following Stella, Jones & Pratchett (2010). Epifaunal invertebrates larger than ~5 cm (e.g., some trapeziids, ophiuroids, and *Alpheus lottini*) were removed with forceps, and smaller invertebrates were removed using a brief rinse (≤60 s) in fresh water without damaging the coral skeleton. Submergence of the corals in fresh water caused epifaunal invertebrates to fall out of the coral branches (e.g., small palaeomonid shrimp). While the validity of this approach has been previously supported (Stella, Jones & Pratchett, 2010), it is possible that some smaller invertebrates may have not been sampled. Invertebrates that escaped from the colonies in the buckets used for transportation were collected by filtering the seawater through a 1 × 1 mm mesh net. The corals were then carefully inspected to ensure collection of all epifaunal invertebrates, including small cryptic macrofauna. Once collected, invertebrates were placed on ice, photographed, fixed in 95% ethanol, and later transported to California State University, Northridge (CA) for identification (and enumeration) (Fig. 1). Following Stella, Jones & Pratchett (2010), each specimen was identified to the lowest possible taxon based on all current literature (e.g., Zootaxa; Castro, Ng & Ahyong, 2004; McKeon & Moore, 2014; Rouzé et al., 2017) and with the assistance of taxonomic consultations. More specifically, 62% of individuals were identified at species level, 18% at genus level and 16% at family or class level. It is possible that ethanol preservation skewed the ability to identify taxa to a finer taxonomic resolution, particularly the palaemonid shrimp.

Several coral colony measurements were taken following Stella, Jones & Pratchett (2010) to test how morphological features including size of coral host affect abundance and diversity of epifaunal invertebrates. For each colony, maximum planar diameter, colony height, space between branches (*n* = 5 random points per colony), penetration depth between branches (*n* = 3 random points per colony) and percentage of partial mortality (to the nearest 5%) were measured (Stella et al., 2014; Counsell et al., 2018). Partial mortality was visually estimated following Pisapia, Anderson & Pratchett (2016). Dead tissue was overgrown by algal turf in all colonies. However, it was not possible to determine time and/or specific cause of injury from these observations. The average of the maximum planar diameter (D) (cm) and the perpendicular diameter (d) (cm) of each colony was used to estimate their two-dimensional projected surface following Linares, Pratchett & Coker (2011). The area was calculated based on the assumption that the colony was an ellipse (A = π* (D and d/2) ^2). Living area (e.g., colony surface area (cm^2^)) was then calculated by multiplying the two-dimensional surface area by 1—proportion of dead tissue for each colony. All remaining measurements were taken with a measuring tape and recorded to the nearest mm. Coral colonies were sacrificed at the end of the study. Research was completed under permits issued by the Haut-commissariat de la Republique en Polynesie Francaise (DRRT) (Protocole d’Accueil 2017–2018).

### Data analyses

Since short-term measurements of flow were not expected to drive instantaneous shifts in resident invertebrate communities, flow was treated as a categorical variable (i.e., colonies were categorically grouped as “upstream or downstream”) in all analyses. Individual generalized linear mixed effects (GLMM) were used to test whether there was a relationship between flow (upstream versus downstream) with each of the five colony characteristics (2D projected surface area, colony height, space between branches, penetration depth between branches and partial mortality). Colony characteristics were the dependent variables and transect was a random variable. Individual GLMMs were then used to test the relationship between colony metrics (colony morphology, colony size and partial host mortality) and flow (upstream versus downstream) on species diversity or abundance. Univariate analyses were chosen to deal with co-linearity between variables and avoid over-parameterized models with insufficient data to estimate coefficients robustly. All assumptions such as goodness of fit, dispersion, and collinearity among the predictors were tested and met. To address potential non-independence issues, the random variable ‘transect’ was included to account for the grouping of coral colonies along the three transects. A Laplacian approximation GLMM with negative binomial distribution was used for the abundance of epifauna model due to over-dispersion of data, while a Poisson distribution was used for the taxon richness model.

We used a PERMANOVA (999 permutations) to investigate potential differences in community structure in decapods (as the dominant functional group; Table 1) associated with varying degrees of partial mortality because live coral tissue is a food source for some decapod species. One colony with 15% of partial mortality was overly influential on statistical analyses because of the lack of data in this category and was excluded from all analyses.

**Table 1:** Total taxon abundances found in *Pocillopora* spp. in the back-reef of Moorea. The unknown Palaemonids were included in abundance but not diversity estimates. Decapoda was the dominant functional group, hence life histories traits are reported based on Huber & Coles (1986), Vytopil & Willis (2001), Stella, Jones & Pratchett (2010) and Stella et al. (2011).

Phylum	Class	Order	Family	Genus and Species	Total Number of individuals	Life-history
Arthropoda	Malacostraca	Decapoda	Trapeziidae	*Trapezia areolata*	4	Specialist
				*Trapezia globosa*	3	Specialist
				*Trapezia formosa*	4	Specialist
				*Trapezia lutea*	100	Specialist
				*Trapezia punctimanus*	2	Specialist
				*Trapezia septata*	1	Specialist
				*Trapezia serenei*	43	Specialist
				*Trapezia tigrina*	8	Specialist
			Alpheidae	*Alpheus lottini*	66	Specialist
				*Alpheus* sp. *White*	10	Specialist
				*Acanthanas* sp.	3	Specialist
				*Synalpheus* sp.	2	Specialist
			Xanthidae	*Chlorodiella nigra*	16	Generalist
				*Chlorodiella* sp.	1	Generalist
				*Psaumis cavipes*	1	Generalist
				*Luniella pugil*	24	Generalist
				*Pilodius* sp.	3	Generalist
				*Liomera cinctimanus*	1	Generalist
			Palaeomonidae	*Coralliocaris*	7	Generalist
				*Harpiliopsis* sp.	7	Generalist
				Unknown	62	Generalist
				*Jocaste* sp.	5	Generalist
			Paguridae	*Paguris* sp.	1	Generalist
			Majiidae	Unknown	1	Generalist
				*Menathius* sp.	2	Generalist
			Epialtidae	*Tiarinia* sp.	4	Generalist
			Diogenidae	*Calcinus* sp.	9	Generalist
			Domeciidae	*Domecia hispida*	12	Generalist
				*Domecia*	2	Generalist
		Tanaidacea		Unknown	1	
		Amphipoda		*Amphipoda*	3	
Echinodermata				*Ophiuroids*	15	
				*Ophiocoma erinaceus*	5	
				*Ophiocoma* sp.	3	
				*Ophiactis* sp.	5	
	Echinoidea	Camarodonta	Echinometridae	*Echinometra* sp.	1	
Mollusca	Bivalvia	Pectinida	Pectinidae	*Chlamys* sp.	1	
	Gastropoda	Neogastropoda	Conidae	*Conus* sp.	1	
			Buccinidae	Unknown	2	
			Marginellidae	*Granulina (margaritula)*	1	
		Caenogastropoda	Cerithidae	*Cerithium litteratum*	1	
				*Cerithium* sp.	4	
			Muricidae	*Coralliophila monodonta*	2	
				Unknown	1	
				*Drupella*	1	
			Littorinimorpha	Cf. *Margitrombus marginatus*	1	
				*Gastropod, glasslike shell*	1	
				Unknown shell	1	
Annelida	Polychaeta			*Chrysopetalum* Sp.	3	
				*Polychaeta*	14	
Chordata	Actinopterygii	Perciformes	Labridae	Unknown	1	
			Gobiidae	*Eviota* sp.	1	

Because taxon richness levels are highly dependent on sample size, we used rarefaction curves to assess any potential underestimation of taxon richness due to a low sample size (Fig. S1). More specifically, using the R package *vegan* and the *rarefy* function, we tested whether the appropriate sample size to measure the community was reached. Rarefaction was performed using counts of individual taxa. Rarefaction curves estimated the expected number of species in a small sample of individuals drawn randomly from a larger sample (Gotelli & Abele, 1983). All analyses were performed using R 3.4.1 packages *lme4*, *vegan*, *stats* and *Mass* (Oksanen et al., 2013; Team et al., 2015).

## Results

A total of 470 individuals belonging to 4 phyla, 16 families, 39 genera, and 18 species were identified (Table 1) from 36 *Pocillopora* spp. colonies. Epifaunal invertebrate densities ranged from 0 to 90 individuals per colony, while taxon richness per colony varied from 2 to 14 (Table 1). Decapod crustaceans comprised the highest proportion (85%) of total epifauna for *Pocillopora* spp. (Table 1). Decapoda mainly belonged to the Trapeziidae, Alpheidae and Palaemonidae (Table 1). Among *Trapezia* crabs, *T. lutea* were the most abundant species living in *Pocillopora* spp. accounting for 25% of all Decapoda (Table 1).

Coral colony areas ranged from 136 to 511 cm^2^ (Table 2). The maximum diameter of coral colonies varied from 11 cm to 24 cm with overall average of 16.9 ± 0.5 cm. Partial mortality was <5% in 19 colonies, 10% in 5 colonies, and 15% in one colony (Table 2). In the absence of any major disturbances, these levels of partial mortality could be considered natural (Wakeford, Done & Johnson, 2008; Pisapia, Anderson & Pratchett, 2016).

**Table 2:** Colony characteristics. Ranges, means and standard errors of each colony characteristics in the back reef of Moorea.

Colony characteristics	Range	Mean	SE
Colony height (cm)	5–27	15.1	0.76
Partial mortality (%)	0–15	3.3	0.69
Surface area (cm^2^)	136–510.7	265.5	14.99
Space between branches (mm)	1.6–3.1	2.1	0.05
Penetration depth between branches (mm)	2.3–8	4.5	0.24

**Figure 2:**
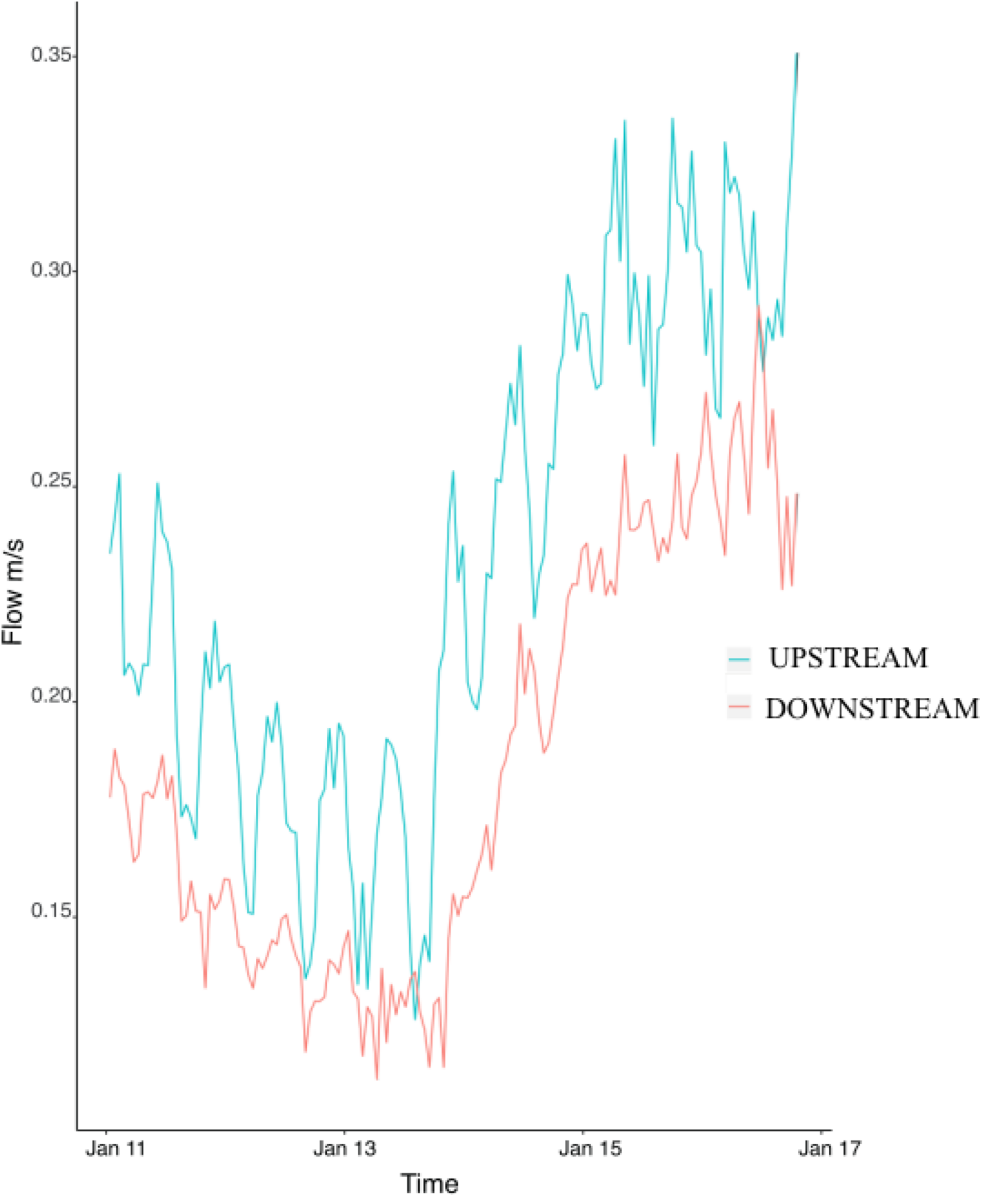
Hourly values of flow speed (m/s) at the upstream mooring (blue line) and the downstream mooring (red line) from the 11th to the 17th of January 2018.

Flow speed at the upstream mooring averaged 0.23 ± 0.004 m/s during the study and was consistently higher than flow at the downstream mooring (0.18 ± 0.004) (Fig. 2). The difference between the upstream and downstream flow velocity was consistent during the six days of sampling (Fig. 2). Flow (i.e., upstream versus downstream) did not have any significant effect on colony characteristics, epifaunal abundance, or richness (Table 3). None of the host characteristics were a good predictor of epifaunal taxon richness and abundance (Table 3). There was also no clear relationship between epifaunal communities and colony size (Fig. S1).

**Table 3:** Summary output of the Generalized Linear Mixed Models (GLMM) for epifaunal abundance and taxon richness. Transect was a random variable. Due to the low replicate number, variation explained by the random effect and the standard deviation equaled to zero or was very low in some models. The dfs were 31. One colony was removed from all analyses.

	Estimate	Standard error	*z*-Value	Pr	Standard deviation (random effect)
**Epifaunal abundance**
Surface area	0.003	0.001	1.05	0.29	1.88 e^−06^
Partial mortality	0.01	0.03	0.40	0.68	0.06
Colony height	0.02	0.32	0.43	0.65	0
Space between branches	0.03	0.42	0.07	0.93	0.07
Penetration depth	0.02	0.07	0.30	0.75	0.07
Flow	0.02	0.22	0.12	0.89	0.07
**Taxon richness**					
Surface area	−0.0003	**<**0.001	−0.41	0.67	0.12
Partial mortality	0.03	0.02	1.70	0.08	0.16
Colony height	0.01	0.01	0.89	0.37	0.11
Space between branches	−0.008	0.24	−0.03	0.91	0.16
Penetration depth	0.05	0.05	0.99	0.32	0.16
Flow	−0.10	0.14	−0.71	0.47	0.16

**Figure 3:**
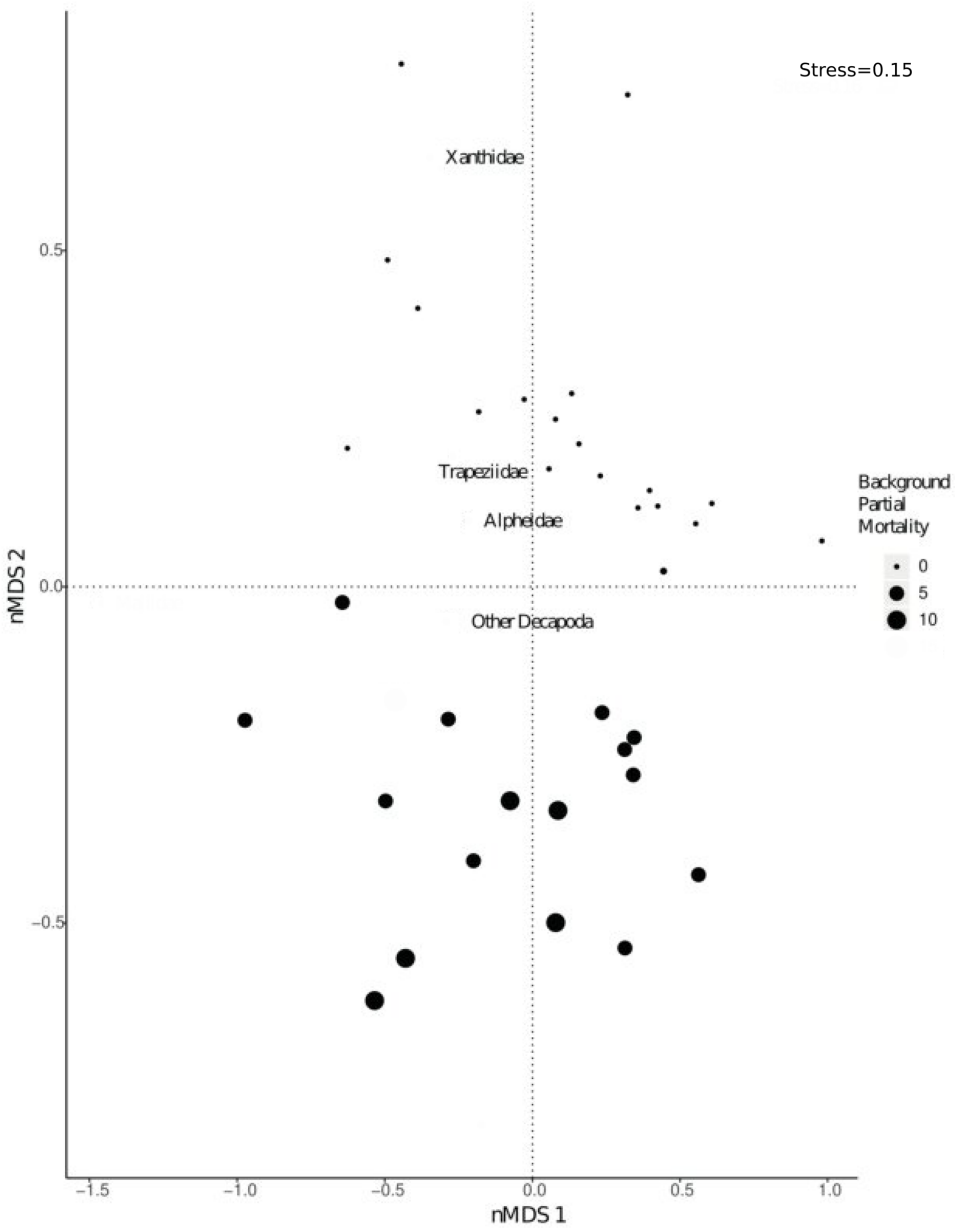
nMDS visually representing PERMANOVA output. PERMANOVA investigated shifts in community composition of Decapoda with varying levels of background partial morality. The nMDS was based on a distance matrix using the counts of each Decapoda taxa in each coral colony. Size of the dots refers to varying percentages of background partial morality.

There was a significant shift in community composition of epifaunal assemblages with different degrees of background partial mortality (PERMANOVA 999 permutations, *F*_1/33_= 21.9, *p* = 0.001; Fig. 3). While obligate live-coral associated taxa, such as *Trapezia* and *Alpheus* (Huber & Coles, 1986; Vytopil & Willis, 2001; Stella, Jones & Pratchett, 2010; Stella et al., 2011) and more generalist Decapoda taxa (Vytopil & Willis, 2001; Stella, Jones & Pratchett, 2010; Stella et al., 2011) were all more abundant in colonies with no partial mortality (Fig. S2), there was not a statistically significant relationship (Fig. S2).

The rarefaction curves indicated a possible underestimation of taxon richness (e.g., the species accumulation curves using corals as samples did not saturate to the regional richness) (Fig. S3). Consequently, an increase in the sample size (e.g., number of coral colonies sampled) may result in an increase in taxon richness (Sgarbi & Melo, 2018).

## Discussion

This study documented a highly diverse and abundant invertebrate community associated with *Pocillopora* spp. commonly found in the back reef of Moorea. Several microhabitat characteristics and one external environmental factor were tested for their influence on invertebrate diversity and abundance. However, epifaunal assemblages associated with *Pocillopora* spp. were consistent and minimally affected by differences in host characteristics and flow regime. These findings highlighted the importance of total habitat availability (e.g., availability of live coral colonies). Abundance and local distribution of invertebrates have been shown to positively correlate with habitat availability in the Great Barrier Reef (Stella et al., 2011). The complex relationships between coral-dwellers and corals will ultimately determine consequences of habitat loss on invertebrate persistence (Enochs, 2012; Enochs & Manzello, 2012).

The most abundant taxa were the decapod crustaceans (and specifically *Trapezia* crabs), which is consistent with other studies (Vytopil & Willis, 2001; Stewart et al., 2006; Stella, Jones & Pratchett, 2010; Stella et al., 2011; Head et al., 2015). Stewart et al. (2006) reported that 95% of individuals in *Pocillopora* colonies were from the genus *Trapezia*. These crabs are also beneficial to the host coral, providing defense from predators such as crown-of-thorns starfish (Glynn, 1980; Pratchett, 2001).

The levels of background partial mortality observed here were well within the range of Pacific reefs in absence of major disturbances, such as the Indian Ocean and Australia’s Great Barrier Reef (Pisapia et al., 2015; Pisapia, Anderson & Pratchett, 2016). Previous findings indicated that partial mortality sustain a diverse range of epifaunal assemblages (Enochs & Hockensmith, 2008; Enochs, 2012; Enochs & Manzello, 2012). However, the percentage of tissue loss in the colonies sampled in this study only ranged between 0% and 15% (Table 2), compared 0–100% reported in Leray et al. (2012). This limited range in tissue loss may explain the lack of effect of tissue loss on epifaunal assemblages.

Different levels of partial mortality corresponded to higher abundance of obligate decapods such as *Trapezia* crabs. However, there was not a statistically significant relationship between abundance of obligate and mutualist Decapoda taxa and partial mortality. Previous studies documented a higher abundance specifically of more obligate taxa such as alpheids and trapeziids in colonies with no partial mortality (Stella, Munday & Jones, 2011; Stella et al., 2011; Leray et al., 2012). Alpheids and trapeziids strongly rely on live corals, have highly specific patterns of coral use (Vytopil & Willis, 2001; Stella, Jones & Pratchett, 2010) and as specialized taxa, they strongly depend on live tissue and are less able to cope with fluctuations in resource availability (Townsend, Begon & Harper, 2003). Xanthids and other generalist decapods were also abundant in colonies with no partial mortality; however, they opportunistically inhabit live coral colonies, and may not be fundamentally dependent upon abundant live coral for their local persistence (Stella, Jones & Pratchett, 2010; Stella et al., 2011). Since obligate coral-dwellers have also been observed in dead colonies (Stella et al., 2011; Head et al., 2015), it is possible that they temporarily move between live and dead colonies, particularly as juveniles, in search of optimal habitat. Importantly, even though dead branches offered sub-optimal habitats, survivorship of obligate coral-dwelling invertebrates will ultimately depend upon availability of live coral tissue (Stella, Jones & Pratchett, 2010; Stella et al., 2011).

Flow at upstream and downstream was not good predictors of abundance and composition of epifaunal invertebrate assemblages on Moorea back reefs. It is possible that at the time of the study differences in flow regime from shore to the reef crest were minimal relative to other times of the year due to high wave-driven swells at the time of the study. During the austral summer, flow on the reef flat is generated by long periods of swell breaking on the reef crest (Hench, Leichter & Monismith, 2008). Even though most of the energy is dissipated when the waves break on the crest, flow speed may still be high across the back reef as observed here (Hench, Leichter & Monismith, 2008; Hench & Rosman, 2013). Previous studies have quantified the importance of physical drivers such as flow, water movement and wave height in driving abundance and diversity of coral assemblages (Gove et al., 2015) and associated epifaunal communities (Counsell et al., 2018).

Small and large coral colonies of Pocilloporids are functionally dissimilar (Edmunds & Burgess, 2016) and this may reflect on diversity and persistence of invertebrate assemblages. While colony size is an important driver of abundance and diversity of coral-dwelling invertebrates (Head et al., 2015; Counsell et al., 2018), findings from the present study indicate that colony size was not related to abundance and taxon richness of these invertebrates. It is possible that the range of host sizes found in this study was relatively small compared to other studies thus explaining a lack of relationships between colony size and epifaunal abundance and taxon richness. Colony size sampled here ranged from 136 to 510.7 cm^2^, the average colony was 16.9 ± 0.5 cm versus 21.1 ± 9.2 cm sampled by Counsell et al. (2018). Within-colony heterogeneity may vary with different colony sizes, for example, larger colonies may have a great variety of light microenvironments across their surface which may or may not be favorable for epifaunal assemblages (Kaniewska et al., 2011; Edmunds & Burgess, 2016). The reduced heterogeneity associated with the small range of host size measured here may explain why there was a weak or no effect of colony size on abundance and taxon richness of epifaunal invertebrates. The correlation between epifaunal abundance and size of dead colonies is also relatively low suggesting that these invertebrates may also not scale proportionally with size of dead colonies (Head et al., 2015).

## Conclusions

This study investigated whether flow regime and host characteristics may affect density and taxon richness of epifaunal assemblages associated with Pocilloporids on a Moorea reef flat. We showed that, for Moorea back reef, epifaunal assemblages associated with *Pocillopora* spp. were consistent and minimally affected by differences in host characteristics and flow regime. The consistency in abundance and taxon richness among colonies (regardless of habitat characteristics) highlighted the importance of total habitat availability. On Moorea back reefs, it is critical to ensure presence of branching corals to support abundance and taxon richness of epifaunal communities.

## Supplemental Information

10.7717/peerj.9364/supp-1 Supplemental Information 1 Relationship between colony size and a) invertebrate abundance and b) taxon richness.

10.7717/peerj.9364/supp-2 Supplemental Information 2 Linear regressions investigating shifts in community composition of Decapoda with varying levels of background partial morality. A) Relationship between partial mortality and obligate decapoda such as trapeziids and alpheids; b) relationship between partial mortality and more generalist decapoda such as Xanthids.

10.7717/peerj.9364/supp-3 Supplemental Information 3 A) Rarefied number of taxa versus the observed number of taxa. B) Rarefaction curves estimating the expected number of taxa in a small sample of individuals drawn randomly from a larger sample. The minimum sample count achieved over the 36 samples was 10. The x-axis refers to the number of samples (coral colonies), while the y-axis refers to number of taxa.

10.7717/peerj.9364/supp-4 Supplemental Information 4 Code used to analyse data and generate graphs.

10.7717/peerj.9364/supp-5 Supplemental Information 5 Raw data.

## Additional Information and Declarations

### Field Study Permissions

The following information was supplied relating to field study approvals (i.e., approving body and any reference numbers): Research was completed under permits issues by the Haut-commissariat de la Republique en Polynesie Francaise (DRRT) (Protocole d’Accueil, 2018).

### Data Availability

The following information was supplied regarding data availability: The raw data and code used in the analyses are available in the Supplemental Files.
