# Supplementary material for: Epifaunal invertebrate assemblages associated with branching Pocilloporids in Moorea, French Polynesia
Source: PeerJ. 2020 Jun 19;8:e9364. doi: 10.7717/peerj.9364 (PMC7307568; doi:10.7717/peerj.9364)
Supplement: Supplemental Information 5 [file peerj-08-9364-s005.docx]

| Colony | Transect | Abundance | Taxon richness | Height (cm) | Partial mortality (%) | Surface Area (cm2) | space between branches (mm) | Penetration depth (mm) |
| --- | --- | --- | --- | --- | --- | --- | --- | --- |
| 1 | 1 | 15 | 6 | 10 | 10 | 240.5 | 2.2 | 3 |
| 2 | 1 | 19 | 14 | 14 | 5 | 165.1 | 2.2 | 3.2 |
| 3 | 1 | 7 | 3 | 15 | 0 | 254.5 | 3.1 | 3.6 |
| 4 | 1 | 15 | 6 | 15 | 5 | 283.5 | 2.6 | 8 |
| 5 | 1 | 23 | 12 | 12 | 10 | 254.5 | 1.9 | 5 |
| 6 | 1 | 4 | 3 | 21 | 0 | 510.7 | 2.7 | 5.1 |
| 7 | 1 | 21 | 10 | 20 | 0 | 165.1 | 2.4 | 4.8 |
| 8 | 1 | 8 | 5 | 17 | 5 | 188.7 | 1.8 | 4.2 |
| 9 | 1 | 12 | 3 | 13 | 0 | 188.7 | 1.8 | 2.3 |
| 10 | 1 | 10 | 6 | 18 | 5 | 143.1 | 1.9 | 3.0 |
| 11 | 1 | 9 | 6 | 16 | 0 | 298.6 | 2.2 | 6.7 |
| 12 | 1 | 25 | 8 | 27 | 0 | 471.4 | 2.0 | 5.0 |
| 13 | 2 | 11 | 4 | 14 | 0 | 227 | 2.0 | 3.0 |
| 14 | 2 | 26 | 12 | 13 | 0 | 254.5 | 2.1 | 5.7 |
| 15 | 2 | 7 | 3 | 10 | 0 | 165.1 | 1.7 | 3.7 |
| 16 | 2 | 5 | 2 | 15 | 0 | 240.5 | 1.8 | 5.8 |
| 17 | 2 | 6 | 5 | 14 | 5 | 283.5 | 1.8 | 3.7 |
| 18 | 2 | 0 | 0 | 14 | 0 | 380.1 | 2.0 | 4.3 |
| 19 | 2 | 3 | 3 | 10 | 0 | 227 | 1.9 | 4.7 |
| 20 | 2 | 5 | 4 | 16 | 0 | 188.7 | 1.9 | 5.3 |
| 21 | 2 | 6 | 4 | 14 | 0 | 176.7 | 2.2 | 4.9 |
| 22 | 2 | 12 | 5 | 26 | 0 | 314.2 | 2.1 | 4.5 |
| 23 | 2 | 9 | 3 | 21 | 10 | 314.2 | 2.3 | 5.0 |
| 24 | 2 | 21 | 10 | 12 | 0 | 283.5 | 1.8 | 3.0 |
| 25 | 3 | 12 | 7 | 12 | 10 | 240.5 | 2.6 | 5.3 |
| 26 | 3 | 1 | 1 | 5 | 5 | 176.7 | 2.3 | 2.7 |
| 27 | 3 | 5 | 3 | 10 | 5 | 240.5 | 1.7 | 4.0 |
| 28 | 3 | 20 | 10 | 20 | 5 | 213.8 | 2.1 | 7.0 |
| 29 | 3 | 90 | 11 | 15 | 15 | 452.4 | 1.9 | 7.5 |
| 30 | 3 | 6 | 4 | 17 | 5 | 330.1 | 2.0 | 4.3 |
| 31 | 3 | 7 | 4 | 11 | 0 | 201.1 | 2.3 | 4.3 |
| 32 | 3 | 7 | 3 | 12 | 5 | 201.1 | 1.8 | 4.3 |
| 33 | 3 | 9 | 5 | 15 | 10 | 254.5 | 2.4 | 2.3 |
| 34 | 3 | 21 | 4 | 10 | 0 | 298.6 | 2.0 | 2.6 |
| 35 | 3 | 4 | 2 | 20 | 0 | 415.5 | 2.0 | 6.7 |
| 36 | 3 | 9 | 5 | 20 | 5 | 314.2 | 2.3 | 5.5 |
